# The CALLY Index May Reflect Systemic Inflammatory Burden Rather than Patient-Reported Disease Activity in Ankylosing Spondylitis: A Medical Record-Based Cross-Sectional Study

**DOI:** 10.3390/jcm15093531

**Published:** 2026-05-05

**Authors:** Altuğ Güner, Taner Dandinoğlu, Sümeyye Tuna Güner, İlknur Aykurt Karlıbel

**Affiliations:** 1 Department of Rheumatology, Bursa City Hospital, 16110 Bursa, Türkiye; 2 Department of Physical Medicine and Rehabilitation, Bursa City Hospital, 16110 Bursa, Türkiye; dandinoglu@gmail.com (T.D.); karlibeli@hotmail.com (İ.A.K.); 3 Family Medicine Specialist, 31 No’lu Şevki Ağır Giden Family Health Center, 16200 Bursa, Türkiye; sumeyye.tuna@saglik.gov.tr

**Keywords:** ankylosing spondylitis, CALLY index, systemic inflammation, disease activity, ASDAS, BASDAI, biomarkers

## Abstract

**Background:** Ankylosing spondylitis (AS) is a chronic inflammatory disease in which accurate assessment of disease activity remains challenging. Although composite indices such as BASDAI and ASDAS are widely used, they may not fully capture systemic inflammatory burden. The C-reactive protein–albumin–lymphocyte (CALLY) index is an emerging composite biomarker integrating inflammatory, nutritional, and immunological components. This study aimed to evaluate the association of the CALLY index with disease activity, functional status, and quality of life in AS. **Methods:** This medical record-based cross-sectional study included 65 patients with AS. Disease activity was assessed using BASDAI and ASDAS-ESR, functional status using BASFI, and quality of life using the 12-Item Short Form Health Survey (SF-12). The CALLY index was calculated from serum CRP, albumin levels, and lymphocyte counts. Correlation and multivariable linear regression analyses were performed. **Results:** The mean CALLY index was 58.43 ± 66.20. The index showed moderate negative correlations with ESR and ASDAS-ESR and a positive correlation with lymphocyte count. Its strong inverse correlation with CRP was expected because CRP is part of the formula and was therefore interpreted cautiously. No significant associations were found with BASDAI, BASFI, or SF-12. In multivariable analysis, BMI (β = −0.299, *p* = 0.012) and NSAID use (β = −0.298, *p* = 0.011) were independent predictors. **Conclusions:** The CALLY index was associated mainly with objective inflammatory markers rather than patient-reported outcomes, suggesting a dissociation between biochemical and clinical disease domains in AS. These findings are preliminary and require confirmation in larger longitudinal studies before clinical application.

## 1. Introduction

AS is a chronic, immune-mediated inflammatory disease characterized by axial involvement, progressive structural damage, and functional impairment [1,2]. Accurate assessment of disease activity remains challenging because available instruments do not fully capture all dimensions of disease burden [3].

Currently, disease activity in AS is commonly evaluated using composite indices such as the Bath Ankylosing Spondylitis Disease Activity Index (BASDAI) and the Ankylosing Spondylitis Disease Activity Score (ASDAS) [4,5,6]. BASDAI is entirely patient-reported, whereas ASDAS incorporates objective inflammatory markers such as erythrocyte sedimentation rate (ESR) or C-reactive protein (CRP). Although these measures are clinically useful, subjective symptoms and biochemical inflammation do not always fully overlap [7,8].

In recent years, composite indices derived from routine laboratory parameters have gained increasing attention as integrative markers of systemic inflammation. The CALLY index is a novel biomarker that integrates inflammatory activity (CRP), nutritional and metabolic status (albumin), and immune competence (lymphocyte count) [9,10].

Previous studies have demonstrated the clinical relevance of the CALLY index in inflammatory and autoimmune diseases, including rheumatoid arthritis and primary Sjögren’s syndrome, where it has been associated with disease activity and clinical outcomes [11,12]. However, its role in ankylosing spondylitis remains unclear.

Therefore, the aim of this study was to investigate the association between the CALLY index and disease activity, functional status, and health-related quality of life in patients with ankylosing spondylitis. We hypothesized that the CALLY index would be more closely associated with objective inflammatory markers than with patient-reported outcomes, reflecting systemic inflammatory burden rather than clinical disease perception.

## 2. Materials and Methods

This single-center, cross-sectional study was based on review of existing medical records from the Rheumatology and Physical Medicine and Rehabilitation outpatient clinics of Bursa City Hospital, Turkiye.

The study was designed and reported in accordance with the Strengthening the Reporting of Observational Studies in Epidemiology (STROBE) guidelines [13]. Ethical approval was obtained from the Institutional Review Board of Bursa City Hospital (Approval No: 2026-3/8; Date: 4 February 2026), and the study was conducted in accordance with the Declaration of Helsinki. Due to the medical record-based design and the use of anonymized data, the requirement for informed consent was waived.

**Figure 1:**
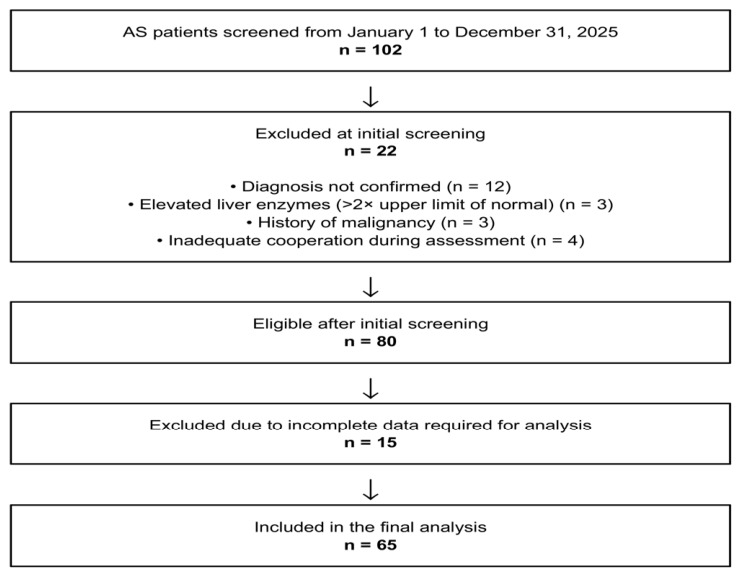
Flow Diagram of Patient Selection and Exclusions.

Patients diagnosed with ankylosing spondylitis according to the modified New York criteria were included in the study [14]. Medical records of patients followed between 1 January 2025 and 31 December 2025 were reviewed. Inclusion criteria were age ≥ 18 years, confirmed diagnosis of ankylosing spondylitis, and availability of complete clinical and laboratory data at a single assessment time point. The patient selection process is summarized in Figure 1. A total of 102 patients with ankylosing spondylitis were screened. After exclusion of 22 patients at the initial screening stage and 15 additional patients due to incomplete data, 65 patients were included in the final analysis. Patients with active or chronic infection, malignancy, advanced hepatic or renal disease, conditions affecting serum albumin levels (such as nephrotic syndrome), or incomplete data were excluded.

Demographic and clinical data, including age, sex, body mass index (BMI), disease duration, medication use (biologic agents and nonsteroidal anti-inflammatory drugs), comorbidities, extra-articular manifestations, and HLA-B27 status, were obtained from electronic medical records. Extra-articular manifestations were identified from the medical records and were considered present if they had been previously documented, regardless of whether they were active at the study visit. Laboratory parameters, including CRP, serum albumin, lymphocyte count, and ESR, were recorded from routine clinical practice.

Disease activity and functional status were assessed using validated indices. BASDAI is a six-item patient-reported instrument scored on a 0–10 numeric rating scale evaluating fatigue, spinal pain, peripheral joint involvement, enthesitis, and both the severity and duration of morning stiffness [4]. ASDAS-ESR is a composite index that integrates both subjective and objective components, including back pain, duration of morning stiffness, patient global assessment, peripheral pain/swelling, and ESR. These components are combined using a weighted formula to generate a continuous score reflecting disease activity. Established cut-off values define disease activity states as inactive disease (<1.3), moderate disease activity (1.3–2.1), high disease activity (2.1–3.5), and very high disease activity (>3.5), allowing standardized categorization of patients [5,6].

Functional status was evaluated using the Bath Ankylosing Spondylitis Functional Index (BASFI), which consists of ten questions assessing the patient’s ability to perform daily activities such as bending, reaching, standing, and climbing steps. Each item is scored on a 0–10 scale, and the final BASFI score is calculated as the mean of all items, with higher scores indicating worse functional impairment. BASFI primarily reflects physical limitations related to spinal mobility and functional capacity and is widely used to assess long-term disability and functional outcomes in patients with AS [15]. BASDAI and BASFI have validated Turkish versions [16,17], whereas ASDAS is a composite index incorporating objective laboratory parameters and therefore does not require language validation [18].

Health-related quality of life was assessed using the validated Turkish version of the SF-12, which evaluates both physical and mental health domains [8,19].

The SF-12 generates two summary scores: the Physical Component Summary (PCS) and the Mental Component Summary (MCS), with higher scores indicating better health status. These components provide a concise assessment of overall health-related quality of life and are widely used in patients with chronic inflammatory diseases. Scores were calculated using standard scoring algorithms.

CALLY index was calculated using the formula (serum albumin × lymphocyte count)/CRP, reflecting an integrated measure of inflammatory, nutritional, and immunological status [9,10]. Given that CRP is included in the CALLY formula, correlations involving CRP were interpreted with caution due to potential mathematical coupling.

Statistical analyses were performed using SPSS v31 Software (IBM Corp., Armork., NY, USA). The distribution of variables was assessed using the Kolmogorov–Smirnov test and confirmed with the Shapiro–Wilk test. Continuous variables were expressed as mean ± standard deviation or median (minimum–maximum), and categorical variables as frequencies and percentages. Group comparisons were performed using the independent samples *t*-test or Mann–Whitney U test as appropriate, and categorical variables were compared using the chi-square test. Correlations between variables were evaluated using Spearman’s rank correlation coefficient. Multiple linear regression analysis with backward elimination was used to identify independent predictors of the CALLY index. Variables with *p* < 0.10 in univariate analysis were included in the initial model, and multicollinearity was assessed using variance inflation factor (VIF), with VIF > 5 considered indicative of collinearity. Model assumptions, including linearity and homoscedasticity, were evaluated using residual plots. A two-tailed *p*-value < 0.05 was considered statistically significant. No formal correction for multiple comparisons was applied.

A post hoc power analysis was conducted using G*Power software (version 3.1) to evaluate the statistical adequacy of the study. For correlation analyses, a two-tailed α of 0.05 and a total sample size of 65 were assumed. The resulting statistical power varied according to the observed effect sizes. The strong correlation between the CALLY index and CRP (*r* = −0.956) corresponded to near-complete power (>0.999), whereas moderate correlations with ESR (*r* = −0.426), ASDAS-ESR (*r* = −0.366), and lymphocyte count (*r* = 0.372) demonstrated adequate power levels of approximately 0.96, 0.87, and 0.88, respectively. In contrast, smaller effect sizes observed for BMI (*r* = −0.293), BASDAI (*r* = −0.183), BASFI (*r* = −0.176), albumin (*r* = 0.206), and SF-12 components were associated with lower statistical power (ranging from 0.29 to 0.67), indicating limited sensitivity to detect weak associations.

For between-group comparisons, the difference in the CALLY index according to NSAID use was associated with a moderate effect size (Cohen’s d = 0.64), corresponding to a power of approximately 0.72, suggesting acceptable but suboptimal statistical power. In contrast, although a large effect size was observed for uveitis (Cohen’s d = 1.19), the marked imbalance between groups (*n* = 62 vs. *n* = 3) resulted in insufficient statistical power (approximately 0.51), thereby limiting the reliability of this finding.

For the multiple linear regression model, the coefficient of determination (R^2^ = 0.241) corresponded to an effect size of f^2^ = 0.318. With four predictor variables and a total sample size of 65, the post hoc power was approximately 0.96, indicating that the model had sufficient power to detect the overall effect. However, the statistical power of individual regression coefficients may be lower, particularly for variables demonstrating borderline significance.

Overall, these findings indicate that the study had adequate statistical power to detect moderate to large effect sizes.

## 3. Results

A total of 65 patients with ankylosing spondylitis (AS) were included in the study. Of these, 16 (24.6%) were female and 49 (75.4%) were male. The mean age was 40.89 ± 10.43 years. The mean BMI was 29.51 ± 6.12 kg/m^2^, and the mean disease duration was 6.89 ± 5.85 years. A total of 46 patients (70.8%) were receiving biological agents, whereas 19 (29.2%) were not. Nonsteroidal anti-inflammatory drugs (NSAIDs) were used by 32 patients (49.2%), while 33 patients (50.8%) were not using NSAIDs. Isoniazid (INH) prophylaxis had been administered to 16 patients (24.6%) and was not administered to 49 patients (75.4%). Comorbidities were present in 20 patients (30.8%), while 45 patients (69.2%) had no comorbid conditions. Regarding extra-articular and related clinical manifestations, psoriasis was observed in 7 patients (10.8%) and uveitis in 3 patients (4.6%). Ulcerative colitis was not observed in any patient. Enthesitis was present in 10 patients (15.4%) and arthritis in 9 patients (13.8%). HLA-B27 positivity was detected in 51 patients (78.5%). The vast majority of patients had axial involvement (64 patients, 98.5%). Laboratory and clinical indices showed a mean C-reactive protein (CRP) level of 7.60 ± 10.05 mg/L, a mean albumin level of 45.89 ± 2.22 g/L, and a mean lymphocyte count of 2.63 ± 1.22 × 10^3^/µL. The mean erythrocyte sedimentation rate (ESR) was 10.20 ± 8.31 mm/h. Disease activity assessment revealed a mean BASDAI score of 4.53 ± 2.20 and a mean ASDAS-ESR score of 2.53 ± 0.97. The mean CALLY index was 58.43 ± 66.20. In terms of health-related quality of life, the mean PCS was 40.67 ± 9.36, and the mean MCS was 42.02 ± 10.94. Additional baseline clinical details are presented in Table 1.

**Figure 2:**
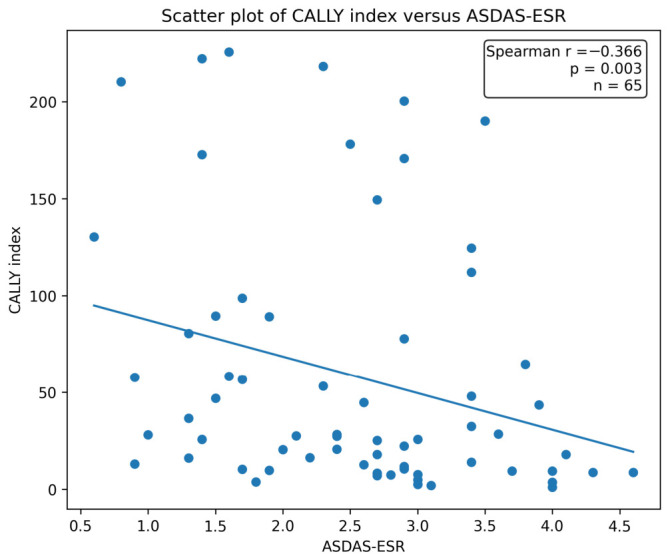
Relationship Between the CALLY Index and ASDAS-ESR in Patients with Ankylosing Spondylitis. A moderate inverse correlation was observed between the CALLY index and ASDAS-ESR (Spearman *r* = −0.366, p = 0.003).

When correlations between the CALLY index and clinical and laboratory variables were examined, no significant association was found with age (*r* = 0.045, *p* = 0.720). Similarly, no significant correlation was observed with disease duration (*r* = 0.033, *p* = 0.792). The CALLY index demonstrated inverse relationships with inflammatory markers. A very strong inverse correlation was observed with C-reactive protein (CRP) (*r* = −0.956, *p* < 0.001); however, because CRP is a direct component of the CALLY formula, this finding was interpreted cautiously. A moderate negative correlation was also observed with erythrocyte sedimentation rate (ESR) (*r* = −0.426, *p* < 0.001). Among disease activity measures, ASDAS-ESR showed a moderate negative correlation with the CALLY index (*r* = −0.366, *p* = 0.003) (Table 2, Figure 2). In contrast, no significant association was found between the CALLY index and BASDAI (*r* = −0.183, *p* = 0.144). A moderate positive correlation was observed between the CALLY index and lymphocyte count (*r* = 0.372, *p* = 0.002). Although a positive association was observed with albumin levels, this relationship did not reach statistical significance (*r* = 0.206, *p* = 0.100). In terms of functional and anthropometric parameters, the CALLY index showed a weak-to-moderate negative correlation with BMI (*r* = −0.293, *p* = 0.018). However, no significant relationship was found with BASFI (*r* = −0.176, *p* = 0.161). Similarly, no association was found with the PCS (*r* = 0.126, *p* = 0.316). Although a positive association was observed with the MCS, it did not reach statistical significance (*r* = 0.232, *p* = 0.063). Overall, beyond the expected CRP-related association arising from mathematical coupling, the CALLY index showed significant relationships with ESR, ASDAS-ESR, lymphocyte count, and BMI, but not with BASDAI, BASFI, or SF-12 component scores. (Table 2).

The CALLY index was compared across various clinical and demographic variables. A statistically significant difference was observed according to NSAID use (*p* = 0.025). Patients not using NSAIDs had a higher CALLY index [78.46 ± 75.79 (median 44.67, range 1.1–225.6)] compared to those using NSAIDs [37.78 ± 47.46 (median 19.22, range 2.1–210.3)]. Among extra-articular manifestations, psoriasis was not associated with a significant difference in the CALLY index (*p* = 0.568). In contrast, uveitis was associated with higher CALLY values (*p* = 0.043); however, because only three patients had uveitis, this finding should be considered exploratory and interpreted with caution. No significant differences were observed according to sex, biologic agent use, INH prophylaxis, comorbidity status, hypertension, coronary artery disease, diabetes mellitus, other comorbidities, psoriasis, enthesitis, arthritis, or HLA-B27 status (Table 3).

To identify independent determinants of the CALLY index, a multiple linear regression analysis was performed using a backward elimination method. The initial model included age, sex, disease duration, BMI, NSAID use, and HLA-B27 status. During the backward elimination process, age (*p* = 0.638) and subsequently disease duration (*p* = 0.353) were removed from the model. The final model retained sex, BMI, NSAID use, and HLA-B27 status. The final model was statistically significant (F = 4.751, *p* = 0.002), with an R^2^ of 0.241 and an adjusted R^2^ of 0.190, indicating that approximately 19.0% of the variance in the CALLY index was explained by the model. In the final model, BMI was independently and inversely associated with the CALLY index (B = −3.238, β = −0.299, *p* = 0.012), indicating that each one-unit increase in BMI was associated with an approximate 3.24-unit decrease in the CALLY index, after adjusting for other variables. Similarly, NSAID use was independently associated with lower CALLY values (B = −39.154, β = −0.298, *p* = 0.011), with NSAID users exhibiting CALLY index values approximately 39.15 units lower compared to non-users. HLA-B27 positivity was associated with higher CALLY index values, with borderline statistical significance (B = 36.194, β = 0.226, *p* = 0.050). Male sex was associated with lower CALLY index values; however, this relationship did not reach statistical significance (B = −32.472, β = −0.213, *p* = 0.072) (Table 4).

## 4. Discussion

In this study, we evaluated the associations between the CALLY index and a broad range of clinical, laboratory, and disease-related parameters in patients with AS. The main findings were that the CALLY index showed a moderate negative correlation with ESR and ASDAS-ESR, while demonstrating a positive correlation with lymphocyte count. In contrast, no significant associations were observed with age, disease duration, BASDAI, BASFI, or quality of life measures. Although a very strong inverse correlation with CRP was also observed, this was expected because CRP is a direct component of the CALLY formula and should not be interpreted as an independent biological finding. These findings suggest a potential dissociation between biochemical inflammatory burden and patient-reported disease domains in AS.

The assessment of disease activity in AS continues to be challenging due to the heterogeneity of clinical manifestations and the limitations of existing indices [1,2]. BASDAI, being entirely patient-reported, may be influenced by subjective factors such as pain perception and comorbidities, whereas ASDAS incorporates objective inflammatory markers and is considered more robust in reflecting inflammatory disease activity [4,5,6]. Previous studies have demonstrated that patient-reported outcomes may not fully align with objective measures of inflammation, supporting the concept of discordance between clinical perception and biological activity [7]. In this context, the observed association between the CALLY index and ASDAS-ESR, but not BASDAI, suggests that the CALLY index is more closely aligned with objective inflammatory burden rather than subjective symptom perception. The mean BASDAI score of 4.53 was slightly above the commonly used threshold for active disease, whereas the mean ASDAS-ESR score of 2.53 indicated moderate-to-high disease activity. This pattern is not unexpected, as BASDAI and ASDAS-ESR, although designed to assess the same clinical construct, do not always classify disease activity in exactly the same way [20,21].

The CALLY index, originally developed as a prognostic marker in oncology, integrates CRP, albumin, and lymphocyte count, thereby reflecting both inflammatory and immunonutritional status [9,10]. Recent studies in rheumatologic diseases have further supported its clinical relevance. In rheumatoid arthritis, the CALLY index has been associated with disease activity and mortality risk, while in primary Sjögren’s syndrome it has shown an inverse relationship with systemic disease activity [11,12].

The very strong inverse correlation between the CALLY index and CRP is expected, as CRP is a direct component of the index. Therefore, this relationship should be interpreted cautiously, as it may largely reflect structural coupling inherent to the index composition rather than an independent biological association. In this context, the associations with ESR and ASDAS-ESR may be more informative than the CRP correlation itself when interpreting the relationship between the CALLY index and inflammatory burden.

The positive association between the CALLY index and lymphocyte count is consistent with its conceptual basis, reflecting immune competence. Although a positive association was also observed with albumin levels, this relationship did not reach statistical significance. The observed inverse association with BMI suggests a potential link between metabolic status and systemic inflammation, as obesity is known to contribute to chronic low-grade inflammatory states.

Importantly, no significant associations were observed between the CALLY index and health-related quality of life as assessed by SF-12 components. This finding suggests that the CALLY index, while reflective of objective inflammatory burden, may not fully capture patient-perceived health status or functional well-being. In rheumatologic diseases, quality of life is a multidimensional construct influenced not only by inflammatory activity but also by pain, functional limitation, fatigue, psychological factors, and comorbid conditions [7,8]. Accordingly, patient-reported outcomes often show only partial overlap with biological disease activity. Our findings support this framework, indicating that immuno-inflammatory burden alone may not fully explain variability in quality of life in AS. These findings support the use of multidimensional assessment strategies that consider both biological and patient-centered measures.

When clinical subgroups were analyzed, differences in the CALLY index were observed for NSAID use and uveitis. Patients not receiving NSAIDs had higher CALLY values, which may reflect differences in inflammatory burden or treatment indication. This association may also reflect confounding by indication, whereby patients with higher inflammatory burden are more likely to receive NSAIDs, potentially influencing the observed relationship and reflecting underlying differences in inflammatory burden. Similarly, the higher CALLY values observed in patients with uveitis may suggest a potential association, although this finding should be considered hypothesis-generating and interpreted with caution due to the very small number of affected patients.

In multivariable analysis, BMI and NSAID use emerged as independent negative predictors of the CALLY index, while HLA-B27 positivity showed borderline significance. These findings suggest that metabolic and treatment-related factors may influence the CALLY index alongside inflammatory processes. Nevertheless, the modest explanatory power of the model indicates that additional biological, metabolic, and disease-related factors may contribute to variability in the CALLY index.

Several limitations should be acknowledged. The cross-sectional design precludes causal inference, and the relatively small sample size, particularly in subgroup analyses, may limit statistical power. In addition, the inclusion of CRP in the CALLY formula introduces potential mathematical coupling. No formal correction for multiple comparisons was applied, which may increase the risk of type I error. Additionally, the lack of external validation limits the generalizability of these findings. Despite adherence to methodological standards, residual confounding cannot be excluded. Therefore, the findings should be interpreted cautiously and considered hypothesis-generating rather than confirmatory.

## 5. Conclusions

In this cross-sectional cohort, the CALLY index appeared to be more closely associated with objective inflammatory parameters than with subjective disease activity or functional status in patients with AS. Its observed associations with CRP, ESR, ASDAS-ESR, lymphocyte count, and BMI suggest that it may reflect inflammatory and immunonutritional aspects of the disease; however, these findings should be interpreted cautiously.

Although the strong dependence on CRP necessitates cautious interpretation, the observed associations with other inflammatory and clinical parameters support further investigation of the CALLY index in AS. Whether it provides meaningful information beyond conventional inflammatory markers, particularly CRP alone, remains to be determined.

Future longitudinal studies with larger and externally validated cohorts are needed to clarify the clinical relevance and potential prognostic value of the CALLY index in axial spondyloarthritis. At present, these findings should be considered preliminary and hypothesis-generating rather than directly applicable to disease monitoring or risk stratification.

## Figures and Tables

**Table 1:** Baseline Demographic, Clinical, and Laboratory Characteristics of the Study Population.

*n* = 65		
**Sex** *n* (%)	Female	16 (24.6)
	Male	49 (75.4)
**Age, mean ± SD (Min–Max)**		40.89 ± 10.43 (20–59)
**BMI, kg/m^2^, mean ± SD (Min–Max)**		29.51 ± 6.12 (18.5–55.7)
**Disease duration, years, mean ± SD (Min–Max)**		6.89 ± 5.85 (1–30)
**Biological agent use,** *n* **(%)**	No	19 (29.2)
	Yes	46 (70.8)
**NSAIDs,** *n* **(%)**	No	33 (50.8)
	Yes	32 (49.2)
**INH prophylaxis,** *n* **(%)**	No	49 (75.4)
	Yes	16 (24.6)
**Comorbidity,** *n* **(%)**	No	45 (69.2)
	Yes	20 (30.8)
	Hypertension	12 (18.5)
	Coronary artery disease	6 (9.2)
	Diabetes mellitus	3 (4.6)
	Other comorbidities	18 (27.7)
**Psoriasis,** *n* **(%)**	No	58 (89.2)
	Yes	7 (10.8)
**Ulcerative colitis,** *n* **(%)**	No	65 (100)
**Uveitis,** *n* **(%)**	No	62 (95.4)
	Yes	3 (4.6)
**Enthesitis,** *n* **(%)**	No	55 (84.6)
	Yes	10 (15.4)
**Arthritis,** *n* **(%)**	No	56 (86.2)
	Yes	9 (13.8)
**HLA-B27,** *n* (%)	No	14 (21.5)
	Yes	51 (78.5)
**Axial involvement** *n* (%)	No	1 (1.5)
	Yes	64 (98.5)
**CRP, mg/L, mean ± SD (Min–Max)**		7.60 ± 10.05 (0.50–60)
**Albumin, g/L, mean ± SD (Min–Max)**		45.89 ± 2.22 (41.5–50.9)
**Lymphocyte, ×10^3^/µL, mean ± SD (Min–Max** **)**		2.63 ± 1.22 (1.38–10)
**ESR, mm/h, mean ± SD (Min–Max)**		10.20 ± 8.31 (2–36)
**BASDAI, mean ± SD (Min–Max)**		4.53 ± 2.20 (0.0–8.4)
**ASDAS-ESR, mean ± SD (Min–Max)**		2.53 ± 0.97 (0.6–4.6)
**CALLY index, mean ± SD (Min–Max)**		58.43 ± 66.20 (1.1–225.6)
**Physical Component Score (PCS), mean ± SD (Min–Max)**		40.67 ± 9.36 (24.02–58.83)
**Mental Component Score (MCS), mean ± SD (Min–Max)**		42.02 ± 10.94 (22.12–63.54)

Abbreviations: BMI, body mass index; NSAIDs, nonsteroidal anti-inflammatory drugs; INH, isoniazid; HLA-B27, human leukocyte antigen B27; CRP, C-reactive protein; ESR, erythrocyte sedimentation rate; BASDAI, Bath Ankylosing Spondylitis Disease Activity Index; ASDAS-ESR, Ankylosing Spondylitis Disease Activity Score based on erythrocyte sedimentation rate; CALLY, C-reactive protein–albumin–lymphocyte index; PCS, Physical Component Summary; MCS, Mental Component Summary. Continuous variables are presented as mean ± standard deviation (minimum–maximum), and categorical variables as *n* (%).

**Table 2:** Correlation Between the CALLY Index and Clinical and Laboratory Parameters.

Variable	CALLY Index	
	*r*	*p*
Age	0.045	0.720
Disease duration, years	0.033	0.792
CRP, mg/L	−0.956	˂0.001
Albumin, g/L	0.206	0.100
Lymphocyte, ×10^3^/µL	0.372	0.002
ESR, mm/h	−0.426	˂0.001
ASDAS-ESR	−0.366	0.003
BASDAI	−0.183	0.144
BASFI	−0.176	0.161
BMI, kg/m^2^	−0.293	0.018
Physical Component Summary (PCS)	0.126	0.316
Mental Component Summary (MCS)	0.232	0.063

Abbreviations: CALLY, C-reactive protein–albumin–lymphocyte index; CRP, C-reactive protein; ESR, erythrocyte sedimentation rate; ASDAS-ESR, Ankylosing Spondylitis Disease Activity Score based on erythrocyte sedimentation rate; BASDAI, Bath Ankylosing Spondylitis Disease Activity Index; BASFI, Bath Ankylosing Spondylitis Functional Index; BMI, body mass index; PCS, Physical Component Summary; MCS, Mental Component Summary. *r*, Spearman’s correlation coefficient.

**Table 3:** Comparison of CALLY Index Values According to Clinical Characteristics.

Variable	Category	Mean ± SD/Median (Min–Max)	*p*-Value
Sex	Female	75.81 ± 70.48/50.90 (2.1–222.3)	0.180
	Male	52.76 ± 64.48/25.23 (1.1–225.6)	
Biological agent	No	43.87 ± 41.95/25.69 (2.1–149.4)	0.840
	Yes	64.45 ± 73.51/28.28 (1.1–225.6)	
NSAIDs	No	78.46 ± 75.79/44.67 (1.1–225.6)	0.025
	Yes	37.78 ± 47.46/19.22 (2.1–210.3)	
INH prophylaxis	Not received	65.51 ± 71.24/27.42 (1.1–225.6)	0.730
	Received	36.76 ± 42.35/27.83 (3.7–178.2)	
Comorbidity	No	57.58 ± 65.97/27.42 (2.1–225.6)	0.853
	Yes	60.36 ± 68.41/27.83 (1.1–222.3)	
Hypertension	No	53.77 ± 60.66/27.42 (1.1–225.6)	0.499
	Yes	79.04 ± 86.82/27.98 (3.7–222.3)	
Coronary artery disease	No	59.22 ± 66.74/28.11 (1.1–225.6)	0.768
	Yes	50.70 ± 66.02/24.07 (3.8–178.2)	
Diabetes mellitus	No	56.30 ± 64.26/27.48 (1.1–225.6)	0.223
	Yes	102.47 ± 106.05/64.57 (20.6–222.3)	
Other comorbidities	No	56.17 ± 62.53/27.55 (2.1–225.6)	0.872
	Yes	64.33 ± 76.61/26.90 (1.1–222.3)	
Psoriasis	No	57.21 ± 63.59/27.83 (1.1–225.6)	0.568
	Yes	68.55 ± 90.59/9.49 (3.8–222.3)	
Uveitis	No	54.88 ± 64.57/26.58 (1.1–225.6)	0.043
	Yes	131.78 ± 67.95/130.35 (64.6–200.4)	
Enthesitis	No	57.25 ± 65.83/27.42 (1.1–225.6)	0.884
	Yes	64.93 ± 71.52/52.60 (2.1–222.3)	
Arthritis	No	59.54 ± 66.25/27.48 (1.1–225.6)	0.621
	Yes	51.52 ± 69.47/28.40 (2.1–222.3)	
HLA-B27	No	35.75 ± 42.79/27.83 (1.1–172.7)	0.389
	Yes	64.66 ± 70.36/27.42 (2.1–225.6)	

Abbreviations: CALLY, C-reactive protein–albumin–lymphocyte index; NSAIDs, nonsteroidal anti-inflammatory drugs; INH, isoniazid; HLA-B27, human leukocyte antigen B27. Data are presented as mean ± standard deviation and median (minimum–maximum).

**Table 4:** Backward Multiple Linear Regression Analysis of Factors Associated with the CALLY Index.

Variable	B	Beta	*p*-Value
Constant	169.332	—	—
Sex (Male)	−32.472	−0.213	0.072
BMI (kg/m^2^)	−3.238	−0.299	0.012
NSAID use (Yes)	−39.154	−0.298	0.011
HLA-B27 positivity (Yes)	36.194	0.226	0.050

Abbreviations: BMI, body mass index; NSAID, nonsteroidal anti-inflammatory drug; HLA-B27, human leukocyte antigen B27; B, unstandardized regression coefficient; Beta, standardized regression coefficient; R^2^, coefficient of determination. Variables entered into the initial model were age, sex, disease duration, BMI, NSAID use, and HLA-B27 status; backward elimination was applied. Final model statistics: *F* = 4.751, *p* = 0.002; R^2^ = 0.241; adjusted R^2^ = 0.190.

## Data Availability

The data that support the findings of this study are available from the corresponding author upon reasonable request. The data are not publicly available due to privacy and ethical restrictions.

## Abbreviations

The following abbreviations are used in this manuscript:

AS	Ankylosing spondylitis
ASDAS	Ankylosing Spondylitis Disease Activity Score
BASDAI	Bath Ankylosing Spondylitis Disease Activity Index
BASFI	Bath Ankylosing Spondylitis Functional Index
BMI	Body mass index
CALLY	C-reactive protein–albumin–lymphocyte index
CRP	C-reactive protein
ESR	Erythrocyte sedimentation rate
HLA-B27	Human leukocyte antigen B27
MCS	Mental Component Summary
PCS	Physical Component Summary
NSAIDs	Nonsteroidal anti-inflammatory drugs
SF-12	12-Item Short Form Health Survey
